# Quantifying uncertainty: a practical comparison of Bayesian and frequentist meta-analytic frameworks applied to lead exposure and children’s IQ

**DOI:** 10.1186/s12874-026-03010-z

**Published:** 2026-09-24

**Authors:** Paulina Sell, Margaux Sanchez, Philippe Palmont, Simon Steiger, Dietrich Plass

**Affiliations:** 1 Environmental Hygiene, German Environment Agency, Berlin, Germany; 2 French National Agency for Food, Environmental and Occupational Health & Safety, Maisons-Alfort, France; 3 Division of Clinical Epidemiology, Department of Medicine Solna, Karolinska Institutet, Stockholm, Sweden

**Keywords:** Meta-analysis, Evidence synthesis, Bayesian modelling, Uncertainty quantification, Lead exposure, Environmental epidemiology

## Abstract

**Background:**

Uncertainty quantification is central to evidence synthesis for risk assessment and policy-making. Frequentist meta-analysis - currently predominant in epidemiology - quantifies uncertainty through confidence intervals, while Bayesian meta-analysis provides posterior distributions. We performed frequentist and Bayesian meta-analyses to compare uncertainty quantification and applicability for risk assessment using lead exposure and children’s IQ as a case study.

**Methods:**

We systematically searched PubMed and Scopus for epidemiological studies on blood lead level (BLL) and IQ score in children. After screening, risk of bias (RoB) in the identified studies was assessed and regression coefficients were harmonised to the $\:{\mathrm{l}\mathrm{o}\mathrm{g}}_{e}$ scale. In both meta-analyses, we included the same effect estimates, assumed a multilevel structure and used random-effects models. The Bayesian meta-analysis employed a hierarchical model with weakly-informative priors. Sensitivity analyses examined robustness to RoB and prior specification. $\:{\tau\:}^{2}$ and $\:{I}^{2}$ were estimated as measures of between-study heterogeneity.

**Results:**

Twelve studies were included in the meta-analysis. The frequentist model yielded a pooled estimate of -2.45 (95% confidence interval: -3.82 to -1.08) IQ points per unit increase in $\:{\mathrm{l}\mathrm{o}\mathrm{g}}_{e}$-transformed BLL (µg/dL) and a 95% predictive interval of -6.53 to 1.53. The Bayesian model yielded a mean pooled estimate of -2.28 (95% credible interval: -3.54 to -1.04) and a 95% predictive interval of -6.28 to 1.69. Between-study heterogeneity was substantial (frequentist: $\:{\tau\:}^{2}$ = 3.32, $\:{\mathrm{I}}^{2}$ = 85.0%; Bayesian: posterior median $\:{\tau\:}^{2}$ = 3.26, $\:{\mathrm{I}}^{2}$ = 84.7%). The Bayesian approach provided a full posterior distribution for heterogeneity, directly quantifying uncertainty from between-study variation. Sensitivity analyses showed both meta-analyses were robust to RoB, and Bayesian estimates were insensitive to tested priors. Depending on the specified prior configuration, the Bayesian model was less sensitive to studies reporting substantial uncertainty, resulting in a lower pooled effect compared to the frequentist model. This regularisation is particularly valuable in small meta-analyses, where frequentist heterogeneity estimates can be unstable.

**Conclusions:**

We provide updated pooled estimates for the effect of lead on children’s IQ. The Bayesian framework provides posterior distributions, enabling direct probability statements, e.g. about exceeding risk thresholds, and comparative assessments between pollutants. These features are particularly valuable for risk assessment and policy-making.

**Graphical Abstract:**

**Figure:**
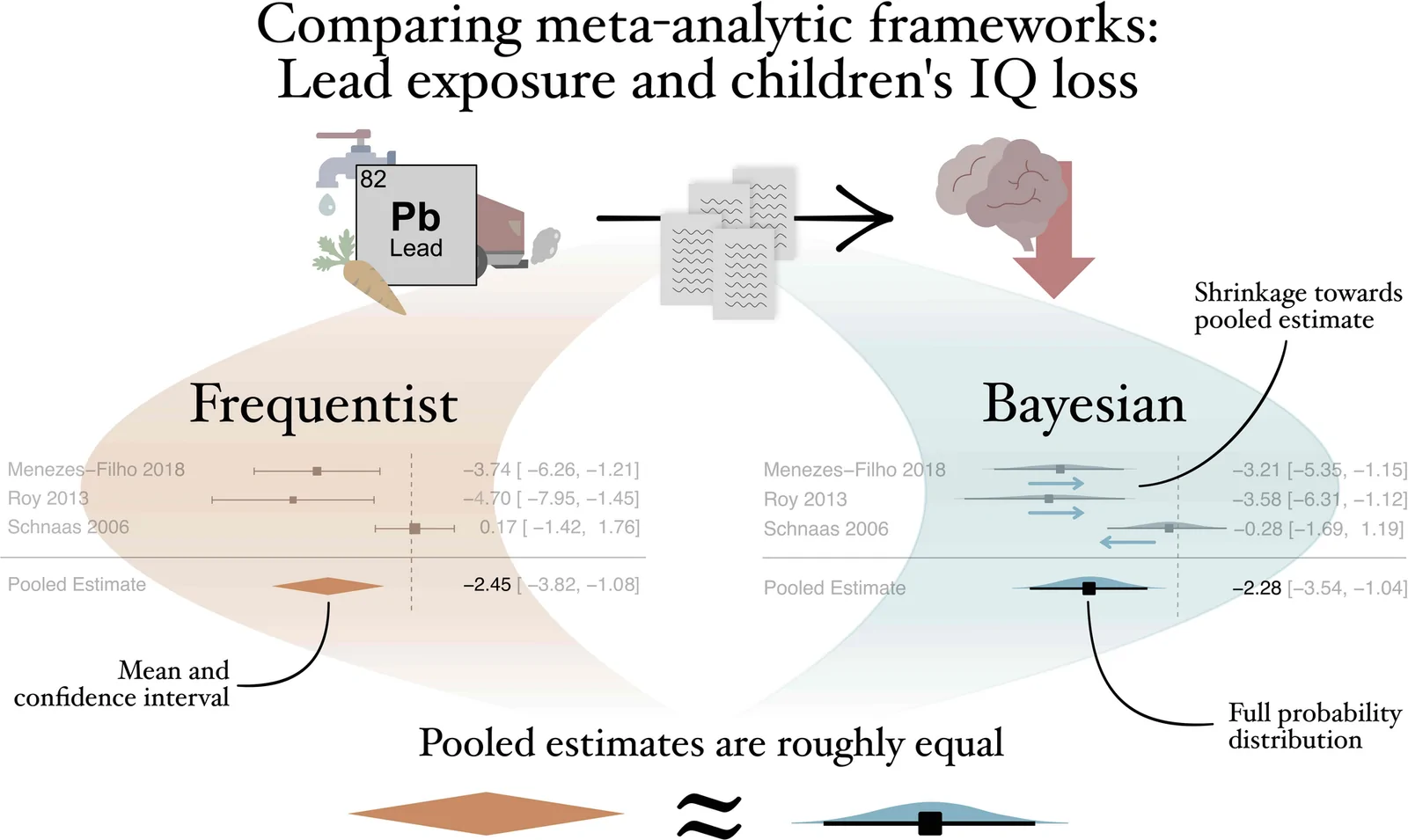

**Supplementary Information:**

The online version contains supplementary material available at https://doi.org/10.1186/s12874-026-03010-z.

## Background

Lead (Pb) is a pervasive environmental pollutant originating from anthropogenic and natural sources. Although concentrations have declined globally in past decades following the phase-out of leaded gasoline, lead remains present in the environment and is detected in human blood samples [1, 2]. In Europe, primary exposure sources include dietary products, drinking water, and indoor dust [3, 4]. The most recent Global Burden of Disease study estimated that in 2023, 3.4 million Disability-Adjusted Life Years (DALYs, 95% uncertainty interval (UI): 2.7 to 4.0 million) were attributable to lead exposure in the European Union (EU) [5]. This underscores the continued public health significance even of low-level exposure.

Exposure to lead is associated with multiple adverse health outcomes across the human life course, including decreased kidney function [6], liver disease [7], coronary artery atherosclerosis [8], cardiovascular mortality [9], infertility in women [10], attention-deficit/hyperactivity disorder in children [11], and antisocial behaviour [12]. Among these endpoints, impaired cognitive development in children — observable as reduced intelligence quotient (IQ) scores — represents a particularly critical endpoint with high public health relevance [13–16]. Children are especially vulnerable to the neurotoxic effects of lead due to their developing nervous systems and relatively high exposure per body mass. Because lead-associated IQ decrements are irreversible and persist throughout life, they lead to substantial and enduring societal costs, making continued efforts to accurately characterise the exposure-response relationship important for evidence-based policy [17].

Blood lead levels (BLL) in the EU have declined markedly and now predominantly fall below 5 µg/dL, well below the concentrations examined in many earlier studies (< 10 µg/dL) [18]. Importantly, there is no evidence for a threshold concentration below which the effects of lead on IQ can be assumed to be negligible [19, 20]. There is evidence for a supralinear exposure-response relationship with steeper IQ decrements per unit increase in BLL at low concentrations compared to higher concentration ranges [16, 21]. These findings highlight the need for an updated quantitative synthesis that reflects both recent epidemiological evidence and the lower exposure range now predominant in European populations, incorporating recent data to inform risk assessment at currently relevant exposure levels.

Existing burden of disease assessments at national and global scales [5, 17, 22–24] have predominantly relied on the foundational study by Lanphear et al. (2005), a pooled analysis of seven cohort studies, or subsequent re-analyses of the same data [16, 25]. While these landmark studies have substantially shaped research and policy on childhood lead exposure, methodological issues and shortcomings have been raised. These include the use of performance IQ instead of full-scale IQ and an erroneous back-transformation of BLL in one cohort in the Lanphear et al. study [25, 26]. These limitations, combined with the availability of more recent epidemiological data, underscore the need not only for an updated evidence synthesis, but also for methodologically robust approaches that can provide reliable exposure-response estimates to be used in burden of disease and risk assessments.

Methodological advances in meta-analysis offer an opportunity to enhance the utility of synthesised estimates for burden of disease and risk assessment applications. While frequentist meta-analysis has been a conventional approach in environmental epidemiology, Bayesian meta-analysis provides a coherent framework for uncertainty quantification through posterior distributions that inherently incorporate both within-study and between-study variability [27]. Unlike frequentist confidence intervals, which are often misinterpreted as having a 95% probability of containing the unknown parameter [28], Bayesian credible intervals directly represent the probability distribution of the parameter of interest, facilitating transparent communication of uncertainty. Furthermore, Bayesian posterior distributions can be directly propagated into probabilistic burden of disease and risk assessments, providing seamless integration with probabilistic frameworks in public health decision-making. By comparing frequentist and Bayesian approaches, we explore how the analytical framework and its approach to uncertainty quantification influence exposure-response estimates and their application.

The aim of this study is to compare uncertainty quantification of frequentist and Bayesian meta-analyses. We apply both meta-analytic approaches to the association between BLL and IQ scores in children using the results of a systematic review on epidemiological studies published between January 2005 and July 2023, thereby also providing an updated pooled effect estimate of this association.

## Methods

### Literature review

This review adheres to the Preferred Reporting Items for Systematic reviews and Meta-Analyses (PRISMA) guidelines [29]. The checklist can be found in additional file 1. The reporting of the Bayesian methods and their results was guided by the Bayesian Analysis Reporting Guidelines (BARG) [30].

We conducted a literature review to identify published studies investigating the association between postnatal (concurrent) exposure to Pb and IQ loss in children. This study was conducted within the EU-funded project *“Partnership for the Assessment of Risks from Chemicals” (PARC)* [31]. The literature review presented here was used in multiple case studies within PARC, including one on the health burden of (methyl)mercury and lead on IQ loss. Here, we will only report results for concurrent lead exposure and IQ loss, but the figures and tables documenting the literature review contain parts of the search for other associations and exposure timeframes. We searched for articles published between January 2005 and July 2023. The year 2005 was chosen because burden of disease studies have predominantly relied on a landmark paper published in that year [16] and an update of the evidence base is therefore warranted, particularly given that lead exposure concentrations have decreased substantially since then.

Search queries in PubMed (MEDLINE) and Scopus included the following terms: substance (Pb - including Medical Subject Headings [MeSH] terms), outcome (IQ and neurodevelopment), population (children) and results (association, exposure-response function [ERF]) (additional file 2).

All titles and abstracts were screened by two reviewers. Any conflicts were resolved by a third person. Full texts were then assessed by one reviewer. Publications were considered relevant according to predefined PECOTS – Population, Exposure, Comparator, Outcome, Timing of exposure and Setting (Table 1).

**Table 1:** PECOTS criteria

PECOTS domain	Inclusion criteria
Population	Human population < 18 years of age (infants, children, teenagers) OR pregnant women and women in childbearing age
Exposure	Exposure to lead and methylmercury through HBM/internal exposure data (urine, blood, hair, bones).
Comparators	Investigating associations between exposure and outcome for single substance or a mixture AND reporting regression coefficient and statistical uncertainty
Outcomes	IQ shift in children < 18
Timing	Current exposure of the children (post-natal) OR current exposure of pregnant women (pre-natal). Both are considered medium- to long-term timing of exposure.
Settings	All settings (location)

*HBM* human biomonitoring

We conducted a rapid update search in PubMed in September 2026, using the same queries as the original search and restricted to records published after July 2023. Titles were first screened by one reviewer and additionally by the large language model (LLM) Claude Opus 5 (Anthropic); when the two judgements deviated, the reviewer revisited the record and made the final decision.

We excluded reviews and background articles that inventoried the literature without quantitatively summarising the results. We also excluded references written in languages other than English. For this present study, only studies that used a continuous, log-transformed BLL scale were included, so that effect estimates could be harmonised. The exposure timeframe for this study was restricted to concurrent, i.e. IQ and BLL measurements must have been conducted at the same time. Descriptive study data was extracted using a grid based on the one proposed by the Office of Health Assessment and Translation within the US National Toxicology Program [32]. For all included studies, effect estimates from the most-adjusted model were extracted and the adjustment set was recorded.

### Risk of bias assessment

To assess study quality, we conducted a risk of bias assessment using the rapid Risk of Bias (RoB) tool with a specific focus on human observational epidemiological studies [33]. Each included publication was assessed for RoB in the following domains: selection, exposure, outcome, confounding, follow-up, analysis, and selective reporting. In total, each study was evaluated using 15 items and scored as either high RoB (-), some concerns (+/-), low RoB (+) or no information (?). If an item was not applicable to the study type (e.g. follow-up in cross-sectional studies), they were marked as “n.a.”. The overall RoB for each individual study was then categorised as either low, moderate or high, following two different summary metrics provided by the RoB tool (for more details, please refer to [33]). Each publication was first assessed by one researcher. Subsequently, consensus with the LLM Claude Sonnet 4.6 (Anthropic) was sought. This was done by uploading the list of all RoB items along with the respective publication. Then, the LLM was prompted to assess the study, using the RoB items. If there were any deviations in the judgement of the researcher and the LLM, the respective item was revisited by the researcher who made the final decision.

### Harmonisation of effect estimates

Studies investigating the association between lead exposure and IQ in children have made different assumptions regarding the shape of the ERF. While some studies assume a linear relationship [34, 35], this assumption may only be valid within very narrow concentration ranges [25]. Studies that tested multiple shapes of the ERF have concluded that a log-linear relationship, where BLL are logarithmically transformed, represents a more appropriate statistical model [25, 36].

The general form of regression model used for a log-linear association is:

1 $$\:{\mathrm{y}}_{pred}=\alpha\:+\beta\:\times\:lo{g}_{b}\left(X\right)+\varepsilon$$

Where $\:{\mathrm{y}}_{pred}$ represents the IQ score, $\:\alpha\:$ is the intercept, $\:\beta\:$ is the regression coefficient (slope), * X* denotes BLL, * b* is the logarithmic base, and $\:\epsilon\:$ is the error term.

In the literature, different logarithmic bases are used to transform BLL, including the natural logarithm ($\:{\mathrm{l}\mathrm{o}\mathrm{g}}_{e}$, where $\:e\approx\:2.718$), base 2, and base 10. To enable pooling of the effect estimates, we harmonised all regression coefficients and their standard error (SE) to increments on the natural logarithm scale ($\:{\mathrm{l}\mathrm{o}\mathrm{g}}_{e}$). The calculations are documented in additional file 3. In some publications, BLL was not on a logarithmic but on an untransformed, linear scale. These were excluded from the meta-analysis, as transformation of effect estimates for linear BLL to log-linear could not be justified.

### Meta-analysis

In order to quantitatively synthesise the available epidemiological literature on the association of children’s blood lead levels and IQ, we conducted separate meta-analyses in the Bayesian and frequentist analytical framework, and compared their results. To make the results of both approaches comparable, both included the same effect estimates from the identified publications, and featured analogous model structure. We estimated pooled effect estimates for the loss of IQ points per continuous unit increase in $\:{\mathrm{l}\mathrm{o}\mathrm{g}}_{e}$-transformed BLL (µg/dL). R version 4.4.2 (2024-10-31) was used for all computations [37]. All code for this study is publicly available on GitHub [38].

#### Bayesian meta-analysis

In the Bayesian framework, we employed a random-effects meta-analysis model. Each study’s observed effect size $\:{\widehat{\theta\:}}_{k}$ is modelled as a noisy reflection of the true underlying effect in that study’s sample $\:{\theta\:}_{k}$, with the noise represented by the within-study sampling error $\:{\sigma\:}_{k}$ (treated as known based on reported study statistics). These study-specific true effects are themselves modelled as draws from an overarching normal distribution which describes the true average effect across studies $\:\mu\:$ and the between-study heterogeneity $\:\tau\:$.

2 $$\begin{aligned}\widehat{\theta}_{k}&\sim\mathcal{N}(\theta_{k},\sigma_{k})\\\theta_{k}&\sim{\mathcal{N}}(\mu,\tau)\end{aligned}$$

Where $\:{\widehat{\theta}}_{k}$ is the observed effect size for study * k*, $\:{\sigma\:}_{k}$ is the within-study sampling standard deviation for study * k*, $\:{\theta\:}_{k}$ is the true underlying effect for study * k*, $\:\mu\:$ is the overall pooled effect and $\:\tau\:$ is the between-study standard deviation (heterogeneity). The Bayesian model was fit using the R package brms version 2.22.0 [39]. Sampling was done using Markov chain Monte Carlo (MCMC). We ran four chains with 4,000 iterations each, of which 2,000 iterations were discarded as warmup, resulting in 8,000 total post-warmup draws. MCMC convergence was assessed using traceplots and the Gelman-Rubin statistic ($\:\widehat{R}$). Chain resolution was assessed using the effective sample size (ESS). Heterogeneity $\:\tau\:$ was directly modelled as a parameter (Eq. 2). $\:{I}^{2}$ was calculated using the typical within-study variance calculated with metafor for each $\:{\tau\:}^{2}$ posterior sample. Because the posterior distribution of $\:{\tau\:}^{2}$ is right-skewed, we summarise $\:{\tau\:}^{2}$ and $\:{I}^{2}$ by their posterior medians rather than their posterior means, as the median represents the typical value more faithfully and keeps the reported point estimates consistent with their credible intervals (for further detail, see additional file 4).

In the Bayesian framework, domain knowledge is elicited and captured as probability distributions (priors) which are subsequently updated by the newly observed data, resulting in a posterior distribution. Prior distributions are specified for all model parameters, i.e. the overall pooled effect estimate $\:\mu\:$ and between-study standard deviation (heterogeneity) $\:\tau\:$. For both parameters, weakly-informative priors were chosen to softly constrain the posterior distributions to realistic ranges of results. The priors are specified on the same scale as the harmonised effect estimates (IQ shift per unit increase in $\:{\mathrm{l}\mathrm{o}\mathrm{g}}_{e}$-transformed BLL).

For the overall pooled effect $\:\mu\:$, a normally distributed prior centred at -1 with a standard deviation (SD) of 2 was chosen. This reflects the prior belief that the overall pooled effect lies between − 5 and 3 with $\:\sim\:$ 95% probability. The centre point of the prior was chosen to be -1 because the negative effects of lead on the developing nervous system and its toxicological mechanisms of action are well established [19]. Since variance terms, such as the between study heterogeneity $\:\tau\:$, must be positive, a truncated normal distribution centred at 0 with a SD of 2 was specified. This reflects the prior belief that heterogeneity $\:\tau\:$ is small to moderate, but does not exclude large heterogeneity if this is present in the included studies. In summary, the following priors were specified for the Bayesian model:

3 $$\begin{aligned}\mu&\sim\mathcal{N}(-1,2)\\\tau&\sim{\mathcal{N}}^{+}(0,2)\end{aligned}$$

Prior predictive checks were conducted using the bayesplot package [40] to verify that the specified priors were appropriate. Posterior predictive checks were carried out to assess if the model structure is able to capture the underlying data well. To quantify uncertainty in the pooled mean and heterogeneity jointly, we constructed the posterior predictive distribution of hypothetical future studies by sampling from $\:\mathcal{N}(\mu\:,\tau\:)$. Additionally, a sensitivity analysis with alternative priors was conducted, as described below.

#### Frequentist meta-analysis

For the frequentist meta-analysis, we employed a random-effects model with a small-sample adjustment, assuming the same multilevel structure as for the Bayesian model (Eq. 2) [41]. Parameters were estimated via the restricted maximum-likelihood (REML) estimation from the R package metafor version 4.8-0 [42]. The $\:{I}^{2}$ statistic was calculated as a measure of heterogeneity, representing the proportion of total variation attributable to between-study heterogeneity $\:{\tau\:}^{2}$. In the frequentist approach, we constructed the predictive distribution of hypothetical future studies via parametric simulation. To enable parametric simulation from point estimates, we assumed a normal distribution for $\:\mu\:$ and a truncated normal distribution for $\:\tau\:$, sampled from these distributions, and then used these samples to draw from the predictive distribution $\:\mathcal{N}(\mu\:,\tau\:)$. The number of draws in the parametric simulation matched the number of MCMC samples from the Bayesian approach.

### Sensitivity analysis

We conducted two sets of sensitivity analyses. To examine the sensitivity of the results to possibly biased study estimates, we ran both the frequentist and Bayesian models again twice, excluding (1) studies with a high RoB rating and (2) with a high or moderate RoB.

To examine the sensitivity of the results to the choice of prior distributions, we fit the Bayesian model again for alternative sets of priors that we consider to be extreme but still reasonable. One set of priors was more informative (narrower) than the main priors, with $\:\mu\:\sim\:\mathcal{N}(-1,1)$ and $\:\tau\:\sim\:{\mathcal{N}}^{+}(0,1)$. A second set of priors was less informative (ignorant) with $\:\mu\:\sim\:\mathcal{N}(0,6)$ and $\:\tau\:\sim\:{\mathcal{N}}^{+}(0,6)$. Note that for $\:\mu\:$, the broader prior is centred at 0, giving equal weight to the probability of a protective and detrimental effect of lead. To be able to identify the individual impact of the prior settings for $\:\mu\:$ and $\:\tau\:$, we varied one of the two parameters while holding the other constant. The location of the $\:\mu\:$ prior was varied to values a third and three times that of the default value, while $\:\tau\:$ was held at $\:{\mathcal{N}}^{+}(0,2)$, and the same was done for the scale of the $\:\tau\:$ prior while holding $\:\mu\:$ at $\:\mathcal{N}(0,100)$ (Table 4). We decided to hold $\:\mu\:$ at $\:\mathcal{N}(0,100)$ instead of using the values from the main analysis $\:\mathcal{N}(-1,2)$ because it allowed us to examine if Bayesian and frequentist estimates converge under a very wide $\:\mu\:$ prior and weak shrinkage.

## Results

### Literature review

The systematic review was conducted with a larger scope and was used as a basis for multiple case studies within the PARC project, which is why it includes another substance (mercury, Hg). The systematic search in PubMed and Scopus yielded 1,073 records for lead. After duplicate removal, title and abstract of 974 records (Pb & Hg) were screened, which left 149 records (Pb & Hg) for full text screening. After this last screening, 36 records remained for Pb. These were then assessed for their eligibility for the meta-analysis. Twelve studies were excluded because they either transformed the BLL variable into categories or left it on a linear (untransformed) scale. Four studies were excluded because they exclusively assessed the effects of prenatal lead exposure. Furthermore, four studies were excluded because the cohorts overlapped with another study and would lead to duplicates. Four additional studies were excluded because they either solely reported effects of chemical mixtures, used non-linear models (including the use of splines) or failed to report essential statistics. Finally, twelve studies were included in the meta-analysis. Study characteristics and regression coefficients (effect estimates) are shown in Table 2.

**Table 2:** Study characteristics

First author, year	Study population (*N*)	Geographical area	Mean age / age range	BLL indicator	BLL value (µg/dL unless stated otherwise)	IQ measurement instrument	Mean IQ	Adjustment set (confounder)	Original BLL scale	Original regression coefficient	Original SE	Harmonized regression coefficient (log(e) scale)	Harmonized SE
Bauer 2020 [43]	635	Province of Bresia, Italy	10-14	Median	1.3	WISC-III (FSIQ)	107.1	Sex, age, SES, hemoglobin, HOME score and HOME score squared	Log(e)	-0.10	0.87	-0.10	0.87
Crump 2013 [25]	1,355	USA (Boston, Cleveland, Cincinnati, Rochester), Mexico, Australia (Port Pirie) and Yugoslavia	5-7	Mean	10.0	WISC (FSIQ)	94.4	Site, HOME score, birth weight, maternal IQ, maternal education, maternal alcohol use, maternal tobacco use and birth order	Log(e)	-3.31	0.63	-3.31	0.63
Dantzer 2020 [44]	224	USA (Greater Cincinnati, Ohio metropolitan region)	12	Mean	0.6	WISC-IV (FSIQ)	101.6	Caregiver FSIQ (WASI-2), BMI at age 12, and a measure of neighborhood-level deprivation based on census-tract level socioeconomic indicators	Log(10)	-13.15	3.71	-5.71	1.61
Desrochers-Couture 2018 [45]	606	Canada (10 different study sites)	3-4	Mean	0.7	WPPSI-III (FSIQ)	107.0	Child age, child sex, evaluation site, marital status, familial income, HOME score, Parenting Stress Index, and cord blood lead (log-2 scale)	Log(2)	0.01	0.04	0.02	0.06
Earl 2016 [46]	127	Australia, New South Wales, Port Pirie and Broken Hill	7-8	Mean	3.9	WISC-IV (FSIQ)	100.5	Current maternal smoking, breastfeeding, income, HOME score and stressful life events	Log(10)	-13.50	5.10	-5.86	2.22
Hong 2015 [47]	839	South Korea (two urban cities, two industrial cities, and one rural district)	8-11	Mean	1.8	Korean WISC (FSIQ)	110.1	Demographic variables and ADHD-RS and CPT scores, plus log10-transformed environmental chemical concentrations (blood mercury and manganese concentrations, and creatinine-standardized urine concentrations of cotinine, phthalate metabolites, and Bisphenol A)	Log(10)	-7.23	3.14	-3.14	1.37
Lee 2021 [48]	502	South Korea (Seoul, Gyeonggi, and Incheon provinces)	6	Mean	1.4	Korean WISC (FSIQ)	109.9	Maternal education, exposure to environmental tobacco smoke during pregnancy, maternal age and maternal IQ	Log(e)	-3.83	2.25	-3.83	2.25
Lucchini 2019 [49]	299	Southern Italy (City of Taranto, Puglia region, 5 sub-areas, incremental distance from a highly industrial area)	6-12	Median	8.4 ng/mL	WISC-IV (FSIQ)	106.0	Age, sex, maternal, Standard Progressive Matrice, non-verbal intellectual efficiency, HOME score and distance from the point source	Log(e) per ng/mL	-1.10	0.84	-1.10	0.84
Lucchini 2012 [50]	299	Northern Italy (Valcamonica and Garda Lake regions)	11-14	Median	1.5	WISC-III (FSIQ)	106.3	SES, area, alcohol consumption, hemoglobin levels	Log(e)	-3.48	1.48	-3.48	1.48
Menezes-Filho 2018 [51]	155	Brazil (Municipality of Simões Filho, metropolitan region of Salvador, Bahia)	7-12	Mean	1.6	WASI (FSIQ)	75.5	Age, maternal IQ and manganese levels	Log(10)	-8.61	2.97	-3.74	1.29
Roy 2013 [52]	708	India (Chennai)	3-7	Mean	11.5	Binet–Kamath Scale of Intelligence (Tamil version of Stanford–Binet) (FSIQ)	106.9	Age, age² in months, sex, mid-arm circumference, average monthly household income, parental education, family size	Log(e)	-4.70	1.66	-4.70	1.66
Schnaas 2006 [36]	150	Mexico (Mexico City)	6-10	Mean	6.7	WISC-Revised (FSIQ)	107.9	Maternal IQ, child sex, birth weight, socioeconomic status (based on parental education, occupation, and income), indicator for first FSIQ testing	Log(e)	0.17	0.81	0.17	0.81

*ADHD-RS* Attention Deficit Hyperactivity Disorder Rating Scale, *BMI* Body Mass Index, *CPT* Continuous Performance Test, *ERF* exposure-response function, *FSIQ* full scale IQ, *HOME* Home Observation for Measurement of the Environment, *SE* standard error, *SES* socioeconomic status, *WASI* Wechsler Abbreviated Scale of Intelligence, *WISC* Wechsler Intelligence Scale for Children, *WPPSI* Wechsler Preschool and Primary Scale of Intelligence

**Fig. 1:**
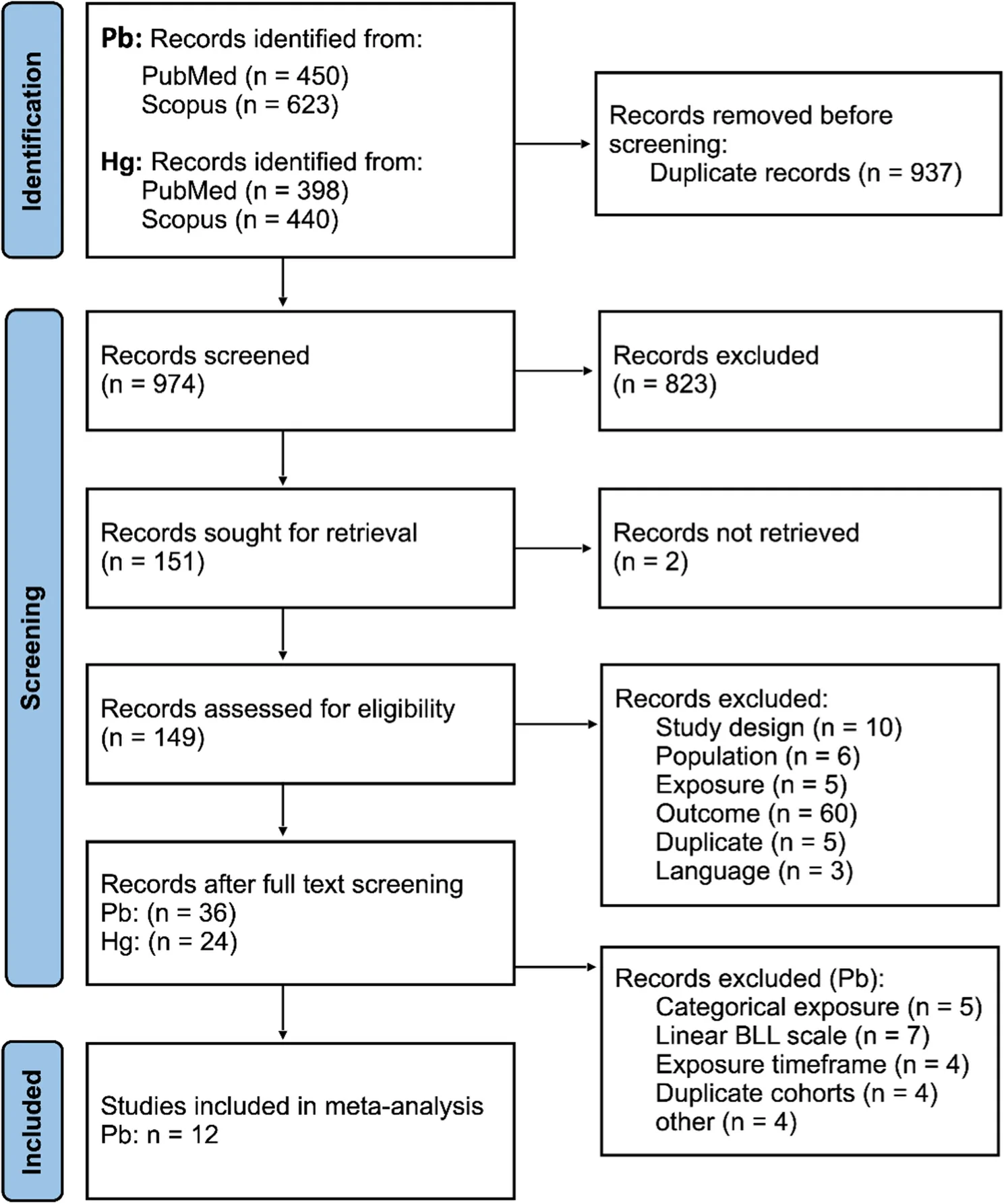
PRISMA flow diagram. BLL, blood lead level; Pb, lead; Hg, mercury

A PRISMA flow diagram summarising the review process is shown in Fig. 1 [29]. Detailed information on the screening process and exclusion reasons can be found in additional file 3.

The size of the study population in included studies ranged from 127 [46] to 1,355 [25]. Study designs included cross-sectional [43, 46, 47, 49–52] and cohort studies [25, 36, 44, 45, 48]. The included studies were conducted in multiple different regions of the world (North America: 4, South America: 1, Europe: 3, Asia: 3, Australia: 1) and some populations were sampled in hotspot regions with presumably higher lead concentrations [43, 46, 51]. The study by Roy et al. [52] reported implausibly large effect sizes. We contacted the corresponding author, who confirmed that decimal points were missing in the publication.

The rapid update search in PubMed yielded 173 records published after July 2023, none of which met the eligibility criteria. 44 and 40 records were excluded because the exposure or outcome definition did not match, respectively; 30 records were excluded because the record was a review rather than a primary study and 59 records were excluded for other reasons, including animal studies and studies on the mechanisms of lead neurotoxicity. Therefore, no new studies were added to the meta-analysis. Screening decisions for the update are documented in additional file 3.

### Risk of bias

The rapid RoB assessment resulted in eight studies with a low RoB rating [36, 43–45, 47, 48, 51, 52], two with a moderate RoB [49, 50] and two studies with a high RoB rating [25, 46]. Among the twelve included studies, items with a high overall RoB were *non-response rate* (7x high RoB, 5x some concerns), where some studies did not report the non-response rate or did not discuss whether non-responding individuals might systematically differ from included participants, leading to potential selection bias. Another item with overall high RoB was *accounting for confounding* (2x high RoB, 5x some concerns), where important confounders like maternal IQ were not included or the authors used a data-driven selection approach for covariate selection, when a theory-driven approach was preferable [53]. Finally, in the item *handling of missing values*, some of the studies contained no information on how missing data was handled, while in other studies they were handled inadequately (3x high RoB, 4x some concerns). Results from the RoB assessment are presented in Table 3.

**Table 3:** Risk of bias assessment

RoB Domain	Selection				Exposure		Outcome		Confounding		Follow-up		Analysis		Selective Reporting	Overall Risk of Bias
RoB Criterion	Eligibility criteria	Comparability of exposure/case and comparison/control groups	Non-response rate	Recruitment time frame	Accuracy, validity and reliability of exposure measurements	Misclassification of exposure	Accuracy, validity and reliability of outcome measurements	Misclassification of outcome	Accounting for confounding	Accuracy, validity and reliability of confounding variables meas.	Adequacy of length of follow-up	Amount and handling of loss to follow-up	Appropriate data analysis methods	Handling of missing values	Selective reporting of outcomes	Assessors judgement of overall bias
Bauer 2020 [43]	+	+/-	-	+	?	+	+	+	+/-	+	n.a.	n.a.	+	+	-	**low**
Crump 2013 [25]	+	+	+/-	-	+/-	-	+	+	-	-	+	+	-	-	+	**high**
Dantzer 2020 [44]	-	+	-	+	+	+	+	+	+	+	+	+	+	+/-	+	**low**
Desrochers-Couture 2018 [45]	+	+	-	+	?	+	+	+	+	+	+/-	+	+	+	+	**low**
Earl 2016 [46]	+	+/-	-	+/-	-	+	+	+	-	+/-	+	?	+/-	-	+/-	**high**
Hong 2015 [47]	+	+	+/-	+	+	+/-	+	+	+/-	+	n.a.	n.a.	+	+	+	**low**
Lee 2021 [48]	+	+	+/-	+	+	+	+	+	+	+	+	+/-	+	+/-	+	**low**
Lucchini 2019 [49]	+	+	-	-	+	+	+	+	+	+	n.a.	n.a.	+	+/-	+	**moderate**
Lucchini 2012 [50]	+	+	+/-	-	+	+	+	+	+/-	+/-	n.a.	n.a.	+	-	+	**moderate**
Menezes-Filho 2018 [51]	+	+	-	+	+	+	+	+	+/-	+	n.a.	n.a.	+	+/-	+	**low**
Roy 2013 [52]	+	+	-	+	+	+	+	+	+/-	+	n.a.	n.a.	+	+	+	**low**
Schnaas 2006 [36]	+	+	+/-	+	+	+/-	+	+	+	+	n.a.	n.a.	+	+	+	**low**

*RoB* risk of bias

(-), high RoB; (+/-), some concerns / moderate; (+), low RoB; (?), no information; (n.a.), not applicable

### Meta-analysis

The prior predictive check indicated that the specified priors were appropriate (additional file 5a). The posterior predictive check showed that the model correctly captured important patterns in the data (additional file 5b). $\:\widehat{R}$ values indicated that all MCMC chains converged (1.00 for both $\:\mu\:$ and $\:\tau\:$). MCMC trace plots showed well-mixed chains and convergence (additional file 5c). ESS indicated that sampling was efficient and central point estimates and credible intervals could be interpreted with confidence (ESS bulk for $\:\mu\:$: 1,796; ESS tail: 2,504, corresponding to 22.5% and 31.3% of total samples respectively).

**Fig. 2:**
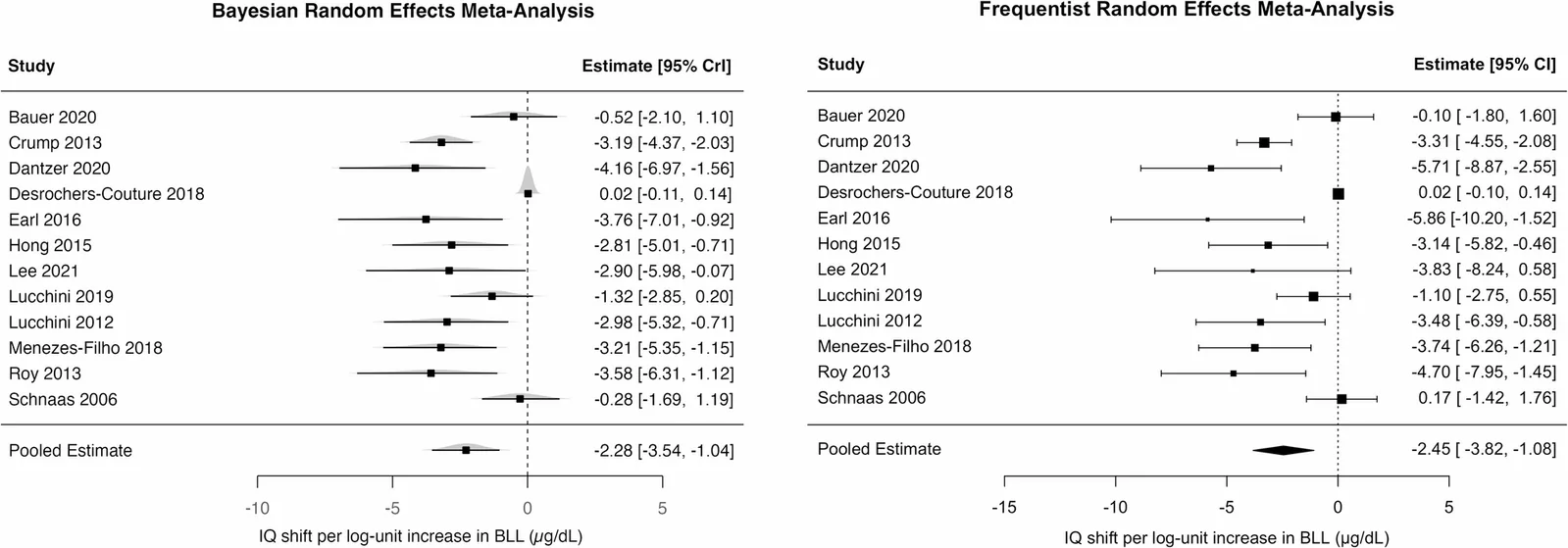
Forest plots for Bayesian (left) and frequentist (right) meta-analyses. BLL, blood lead level; CI, confidence interval; CrI, credible interval

The Bayesian random-effects meta-analysis yielded a posterior mean pooled estimate of -2.28 (95% credible interval (CrI): -3.54 to -1.04) IQ points per log-unit increase in BLL (µg/dL) (see Fig. 2) and a 95% predictive interval from − 6.28 to 1.69. The frequentist approach produced a comparable pooled estimate of -2.45 (95% confidence interval (CI): -3.82 to -1.08) and a comparable 95% predictive interval from − 6.53 to 1.53. Substantial between-study heterogeneity was observed under both frameworks. In the Bayesian model, between-study heterogeneity was estimated as a posterior median $\:{\tau\:}^{2}$ of 3.26 (95% CrI: 1.04 to 9.50), corresponding to an $\:{\mathrm{I}}^{2}$ of 84.7% (95% CrI: 63.9% to 94.2%). The frequentist model estimated a closely comparable $\:{\tau\:}^{2}$ = 3.32 (95% CI: 0.99 to 11.67), with $\:{\mathrm{I}}^{2}$ = 85.0% (95% CI: 62.7% to 95.2%). Additional file 6 contains the full model code and output for both models. The results of both models are shown as forest plots in Fig. 2.

The single study estimates shown in Fig. 2 differ between frameworks because Bayesian shrinkage pulls individual study estimates towards the pooled mean, borrowing information from the overall mean. This has no equivalent in the frequentist framework, where the contribution of each study to the pooled effect is determined by inverse-variance weighting. The degree of shrinkage does, however, depend on each study’s precision in a way that parallels inverse-variance weighting: more precise studies are shrunk less (and are also weighted more heavily towards the frequentist pooled estimate), while less precise studies are shrunk more (and weighted less).

### Sensitivity analysis

#### Risk of bias

For both the frequentist and Bayesian model, the strength of the mean pooled effect decreased after removing studies with high risk of bias and slightly increased again after removing high and moderate RoB studies. These shifts in both directions and their small size showed that the results were robust to possible individual-study bias (Table 4).

**Table 4:** Results of sensitivity analyses by framework

Framework	Variant	Estimate (95% UI)
RoB		
Bayesian	**Full sample**	**-2.28 (-3.54, -1.04)**
	Low RoB only	-1.96 (-3.60, -0.43)
	Low/Medium RoB	-1.95 (-3.39, -0.63)
Frequentist	**Full sample**	**-2.45 (-3.82**,** -1.08)**
	Low RoB only	-2.23 (-4.21, -0.25)
	Low/Medium RoB	-2.14 (-3.68, -0.60)
Priors		
Bayesian	**Main**	**-2.28 (-3.54**,** -1.04)**
	Narrow: mu = N(-1, 1), tau = N+(0, 1)	-2.00 (-3.01, -1.02)
	Ignorant: mu = N(0, 6), tau = N+(0, 6)	-2.46 (-3.97, -1.13)
	Skeptical: mu = N(-0.33, 2), tau = N+(0, 2)	-2.19 (-3.46, -0.96)
	Enthusiastic: mu = N(-3, 2), tau = N+(0, 2)	-2.49 (-3.82, -1.29)
	Default shrinkage: mu = N(0,100), tau = N+(0, 2)	-2.43 (-3.90, -1.14)
	Strong shrinkage: mu = N(0,100), tau = N+(0, 0.67)	-2.26 (-3.42, -1.20)
	Weak shrinkage: mu = N(0,100), tau = N+(0, 6)	-2.49 (-4.04, -1.11)

*RoB* risk of bias, *UI* uncertainty interval (credible interval for Bayesian and confidence interval for frequentist model results)

Configurations varying only the effect prior hold the heterogeneity prior at N+(0, 2); configurations varying only the heterogeneity prior hold the effect prior at Normal(0, 100), which is effectively flat but not improper. N+() denotes a Normal distribution with a lower bound of 0

#### Prior sensitivity

With more informative (narrower) priors ($\:\mu\:\sim\:\mathcal{N}(-1,1)$, $\:\tau\:\sim\:{\mathcal{N}}^{+}(0,1)$), the posterior mean of $\:\mu\:$ decreased (Table 4). This is expected, because the narrow $\:\mu\:$ prior, together with stronger shrinkage, favours a tighter posterior distribution with effect ranges closer to -1, the mean of $\:\mu\:$’s prior. When using less informative (ignorant) priors ($\:\mu\:\sim\:\mathcal{N}(0,6)$, $\:\tau\:\sim\:{\mathcal{N}}^{+}(0,6)$), the posterior mean of $\:\mu\:$ increased slightly compared to the main model. This set of priors gives more weight to studies reporting high effects with large uncertainty ranges.

Varying the prior on the location of $\:\mu\:$ while holding $\:\tau\:$ at $\:{\mathcal{N}}^{+}(0,2)$, we found neither skeptical nor enthusiastic priors on the location of $\:\mu\:$ substantially altered what we concluded from the estimates. Varying the strength of shrinkage by decreasing or increasing the scale of $\:\tau\:$ while holding $\:\mu\:$ at $\:\mathcal{N}(0,100)$ also did not substantially alter our conclusions. The estimate for $\:\mu\:$ under weak shrinkage matched that of the frequentist analysis, while the estimate under strong shrinkage was notably more conservative. This is because many of the strongly negative coefficients were reported by studies with large uncertainty which are in turn more affected by shrinkage, pulling their estimands closer towards zero.

For further detail on these analyses, see additional file 5d-f for prior, likelihood and posterior distributions of all prior sensitivity analyses.

## Discussion

In this study, we compare uncertainty quantification of a frequentist and a Bayesian meta-analysis, which is less used in the field of environmental epidemiology to date, using lead exposure and children’s IQ as an applied example.

While previous publications have compared both meta-analytic frameworks, they focus either on evidence-based medicine [54–57] or network meta-analysis [58, 59] and thus address methodological challenges that only partially overlap with those encountered in environmental epidemiology. To our knowledge, this is the first study to compare Bayesian and frequentist meta-analytic approaches in this field.

Pooled effect estimates, measures of heterogeneity, and predictive intervals were largely consistent across both models, though the Bayesian model yielded slightly lower mean pooled effects. This is attributable to both the prior for the overall effect ($\:\mu\:$) and the posterior shrinkage of individual study estimates, influenced by the heterogeneity prior ($\:\tau\:$), as illustrated by the sensitivity analyses (Table 4). Importantly, this difference does not relevantly affect the overall interpretation of the association between lead exposure and IQ.

The two frameworks may differ in how they treat studies reporting substantial uncertainty depending on prior configuration and sample size. Bayesian meta-analysis can be set up such that it is less sensitive to uncertain studies via the prior distribution for the heterogeneity: the more concentrated this prior is towards zero the stronger the shrinkage, pulling uncertain studies more towards the pooled estimate than certain studies (Table 4). The frequentist meta-analysis can configure the weighting of individual studies by changing the estimation methods for $\:{\tau\:}^{2}$. At the time of writing, the metafor package included a list of references pointing to publications for each of these estimation methods. However, a vignette to illustrate the use of these methods to make them more accessible to applied researchers was not available [60].

Conducting a Bayesian meta-analysis can be more demanding than frequentist approaches, requiring greater statistical knowledge, explicit consideration of modelling assumptions, and typically more programming effort [61]. While these demands may initially appear as disadvantages, they can equally be understood as methodological safeguards: by requiring researchers to state assumptions explicitly rather than accepting software defaults, the Bayesian approach encourages deeper engagement with the data and statistical model.

A key distinction between the two frameworks is in how they handle parameter uncertainty. Frequentist models yield point estimates with confidence intervals, whereas Bayesian models produce full posterior distributions. This offers distinct practical advantages, particularly for risk assessment and policy-making. First, any credible interval can be derived from the posterior distribution to meet the needs of researchers, decision-makers, or other stakeholders. These allow direct probability statements about parameter values, unlike confidence intervals which are often incorrectly interpreted as probability statements about parameters. Second, posterior distributions enable probabilistic comparisons across multiple health risks, such as calculating the probability that one risk exceeds another. When meaningful effect sizes or risk thresholds are defined, exceedance probabilities can be directly obtained from the posterior, providing an intuitive and policy-relevant summary of uncertainty. Posterior distributions together with the specified statistical model can be directly used to generate predictions for theoretical future studies, offering an additional way to characterise between-study heterogeneity. They also enable researchers to estimate the population-level health impact of an exposure under specified conditions. This is known as Environmental Burden of Disease (EBD). In EBD studies, health impacts are quantified using metrics such as DALYs, allowing comparisons across health determinants within and across populations, countries, and time periods. Such assessments integrate both population exposure and the exposure–response relationship [62]. The distributional results of the present study enable probabilistic EBD assessments via Monte Carlo simulations without requiring additional assumptions about the uncertainty distribution of the exposure–response function.

The option to create predictive distributions that can inform EBD studies is, however, not exclusive to the Bayesian approach. In the frequentist framework, they can be obtained using, for example, parametric simulation, nonparametric bootstrap, or the delta method. Each method comes with its own assumptions that may impact the results, requiring the researcher to carefully weigh these decisions. In this study, the small sample size led to convergence issues when attempting to use nonparametric bootstrap. We therefore estimated the predictive interval using parametric simulation because it was feasible in our setting and procedurally similar to the Bayesian approach, making it more comparable. We assumed normal and truncated normal distributions for $\:\mu\:$ and $\:\tau\:$, respectively, to match the Bayesian setup but we could have theoretically assumed any distribution for $\:\tau\:$ whose support is limited to positive real values. If we had instead chosen a log-normal distribution for $\:\tau\:$, the predictive interval would have reached from - 6.74 to 1.74. By assuming distributions for $\:\mu\:$ and $\:\tau\:$ after conditioning on the data, the data cannot inform the assumed distributions during parameter estimation. The Bayesian approach differs in that prior distributions will, given sufficient data, converge to the true distribution so long as that prior places non-zero probability on the true values [63].

Our results are consistent with previous toxicological and epidemiological evidence [13, 15, 16, 19] as well as prior meta-analyses on the effects of lead on IQ. Direct comparison of effect magnitudes is, however, limited due to methodological differences: some studies estimated effects per exposure category [20, 64, 65], while others reported partial $\:\mathrm{r}$ [14] or standardised mean differences [66].

The RoB sensitivity analysis indicated that results were generally robust to individual-study bias. Notably, the study published by Crump et al. [25], which is a re-analysis of the landmark study by Lanphear et al. [16], received a high RoB rating in our assessment. Given that this re-analysis has informed multiple downstream analyses, including EBD assessments, its potential to introduce bias is a concern. The primary driver of the high RoB rating was the use of stepwise backward-elimination regression, a method that has been extensively criticised [53, 67, 68] and is central to the findings of Crump et al. This underscores the value of the updated evidence synthesis provided here.

Substantial heterogeneity was observed in this meta-analysis, likely driven by differences in study populations (size, location, age, and exposure range) and statistical methods, particularly the covariate adjustment sets used. Two studies reporting null effects were conducted in relatively low-exposed populations [43, 45], where limited exposure variation reduces the detectability of an effect. Differences in study location may also contribute: IQ tests not adapted to the target population can result in underestimated scores, which may apply to the study by Menezes-Filho et al. [51]. Additionally, studies reporting larger uncertainty tended to report larger effects, suggesting a possible presence of publication bias (additional file 5 g). The Bayesian approach was less sensitive to estimates from such studies, because the results of individual studies with large uncertainty were shrunk towards the pooled estimate, which is a particular advantage when pooling a small number of studies.

This study has several limitations that should be considered when interpreting the pooled estimates. Our original literature search was limited to January 2005 to July 2023 and was updated in September 2026 through a rapid search in PubMed, which identified no additional eligible studies. Although this update was narrower in scope than the original search, it is unlikely that a full update covering both databases would materially change the pooled estimate. Due to resource constraints, full-text screening and RoB assessment could not be carried out by multiple independent persons. The studies we included in the meta-analysis were heterogeneous with respect to covariate adjustment, exposure range, IQ measurement instrument and cohort characteristics, meaning that the coefficients pooled in this study may not represent exactly the same estimand. Publication bias was assessed through visual inspection only, which cannot exclude its presence. This may have led to overestimation of the pooled effect. We further excluded studies that did not report effect estimates on the logarithmic scale of BLL. This was necessary to ensure interpretability, as combining effect estimates expressed on different scales would render the pooled estimate uninterpretable. However, these limitations did not affect the main objective of this study, which was to to compare uncertainty quantification in frequentist and Bayesian meta analysis in an applied setting. It should be noted that a more comprehensive and general comparison of Bayesian and frequentist meta-analytic approaches would require a simulation study, which we think would be useful to conduct in future research, but was outside the scope of the present study.

This study also has several important strengths. By applying a parallel approach to both meta-analytic models — including the use of identical effect estimates, a multilevel random-effects model, and $\:{I}^{2}$ as a common measure of heterogeneity — we provide a direct comparison of a frequentist and a Bayesian random-effects meta-analysis. We also demonstrate how both approaches quantify uncertainty in predictive quantities, exemplified by calculating the distribution of hypothetical new studies. To our knowledge, this is the first study to formally conduct such a comparison in the field of environmental epidemiology. Beyond this methodological contribution, we provide an evidence synthesis on the association between lead exposure and children’s IQ based on a systematic literature review. All data and code, including the code used to produce this manuscript, are publicly available, enabling other researchers to reproduce and build on our analyses. Finally, we adhered to PRISMA and BARG reporting guidelines, along with the use of the raRoB tool for risk of bias assessment, ensuring consistency and rigour in study selection, data extraction, study quality appraisal, and statistical analysis.

## Conclusions

This study provides an applied methodological comparison of Bayesian and frequentist meta-analysis and an evidence synthesis of epidemiological studies examining the association between lead exposure and children’s IQ. The resulting pooled effect estimates are consistent with previous evidence and reinforce the importance of reducing environmental lead contamination to reduce the public health burden this substance continues to impose.

In the context of our study, comparing both meta-analytic models showed that the Bayesian approach offered distinct advantages for risk assessment and policy-making by fully quantifying uncertainty – especially the ability to incorporate prior knowledge, comprehensively quantify uncertainty, and produce probabilistic outputs that are more directly useable for decision-makers. While the Bayesian framework currently demands greater statistical knowledge and programming effort than the frequentist approach, these barriers are likely to diminish if the framework gains wider adoption and training becomes more accessible. We believe that the benefits gained outweigh the investments made and therefore want to encourage researchers to consider Bayesian methods.

## Supplementary Information

Additional file 1: PRISMA 2020 checklist; Table containing all PRISMA 2020 items and the location where the items are reported.

Additional file 2: Search queries; Full search queries for the systematic review.

Additional file 3: Literature review summary; Summary of the literature review process, incl. details on exclusion reasons and the effect estimate harmonisation and the screening decisions from the update search.

Additional file 4: Calculation of *I*^2^ in the Bayesian Framework; Detailed rationale and description for the calculation of *I*^2^ in the Bayesian meta-analysis.

Additional file 5: Diagnostic plots for the Bayesian model and funnel plot.

Additional file 6: Model outputs; Full model output for frequentist and Bayesian meta-analysis.

## Data Availability

All data and code needed to reproduce the current study are publicly available on GitHub, https://github.com/PaulinaSell/lead-IQ_meta-analysis.

## Abbreviations

BARG: Bayesian Analysis Reporting Guidelines
BLL: Blood lead level
CI: Confidence interval
CrI: Credible interval
DALY: Disability-adjusted life years
EBD: Environmental Burden of Disease
ERF: Exposure-response function
EU: European Union
IQ: Intelligence quotient
LLM: Large language model
PRISMA: Preferred Reporting Items for Systematic reviews and Meta-Analyses
REML: Restricted maximum-likelihood
RoB: Risk of bias
SD: Standard deviation
