# Supplementary material for: Quantifying uncertainty: a practical comparison of Bayesian and frequentist meta-analytic frameworks applied to lead exposure and children’s IQ
Source: BMC Med Res Methodol. 2026 Sep 24;26:209. doi: 10.1186/s12874-026-03010-z (PMC13602614; doi:10.1186/s12874-026-03010-z)
Supplement: Supplementary file 1 [file 12874_2026_3010_MOESM4_ESM.pdf]

# Calculation of $I^2$ in the Bayesian Framework

This supplementary file describes how $I^2$ was calculated for the Bayesian meta-analysis.

## General definition

Higgins & Thompson (2002) define $I^2$ as the proportion of total variance in observed effect sizes that is attributable to between-study heterogeneity:

$$I^2 = \frac{\tau^2}{\tau^2 + v_t}$$

where $\tau^2$ is the between-study variance and $v_t$ is a measure of typical within-study variance across $k$ included studies.

$v_t$ is a weighted summary of all within-study sampling variances $\sigma_k^2$, computed by `metafor` (Viechtbauer 2010) as:

$$v_t = \frac{(k-1)\sum_k w_k}{\left(\sum_k w_k\right)^2 - \sum_k w_k^2}$$

where $w_k = 1/\sigma_k^2$ are the inverse-variance weights.

## Bayesian calculation

As the formula above shows, $v_t$ is calculated from within-study sampling variances $\sigma_k^2$ and therefore not specific to the Bayesian or Frequentist framework. We chose to reuse the value for $v_t$ obtained from `metafor` instead of re-deriving $v_t$ from $\sigma_k^2$, but both approaches would lead to the same result.

We calculated the distribution of $I^2$ by applying the $I^2$ formula from above to each posterior sample of $\tau^2$.

### Mean vs median

We summarised the posterior of $\tau^2$ and $I^2$ using the median rather than the mean, because the median can be applied before or after the conversion from $\tau^2$ to $I^2$ with the same result, whereas the mean cannot. In short, using the median avoids distorting the result, allowing us to compare the Bayesian to the Frequentist estimates.

## References

Higgins, J. P., & Thompson, S. G. Quantifying heterogeneity in a meta-analysis. Statistics in medicine, 2002;21:1539–1558. https://doi.org/10.1002/sim.1186.

Wolfgang Viechtbauer. Conducting meta-analyses in with the metafor package. Journal of Statistical Software 2010;36:1–48. https://doi.org/10.18637/jss.v036.i03.
