# Supplementary material for: Quantifying uncertainty: a practical comparison of Bayesian and frequentist meta-analytic frameworks applied to lead exposure and children’s IQ
Source: BMC Med Res Methodol. 2026 Sep 24;26:209. doi: 10.1186/s12874-026-03010-z (PMC13602614; doi:10.1186/s12874-026-03010-z)
Supplement: Supplementary file 2 [file 12874_2026_3010_MOESM5_ESM.pdf]

Supplementary File 5

This supplementary file shows diagnostic plots for the Bayesian model and a funnel plot.

5a) Prior predictive check

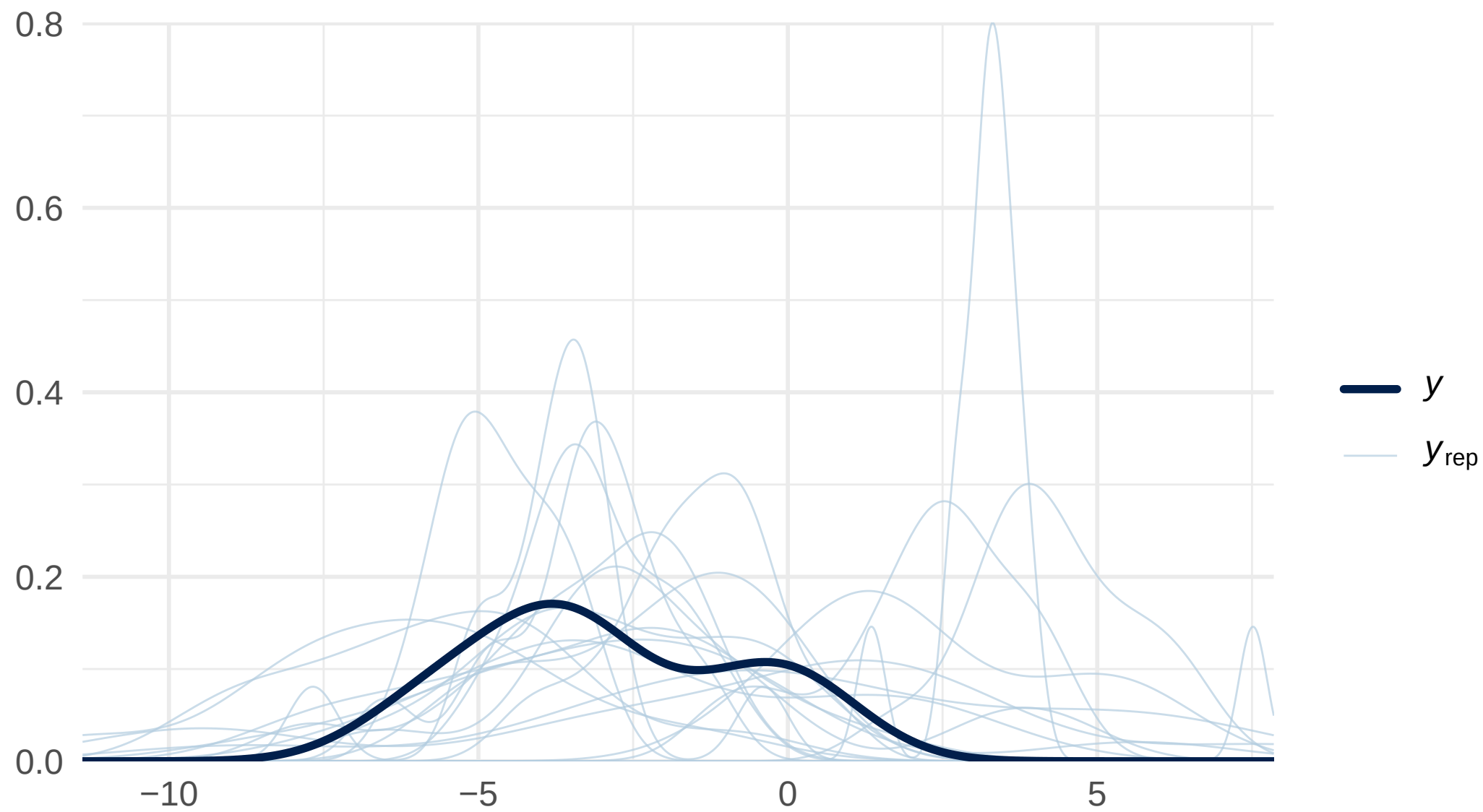

Prior predictive check, using `pp_check()` from the bayesplot package. Density of the observed effect sizes (dark line) compared with 20 data sets simulated from the prior predictive distribution (light lines), obtained by fitting the model with sample_prior = "only". This checks whether the priors imply plausible effect sizes before the observed data are taken into account.

5b) Posterior predictive check

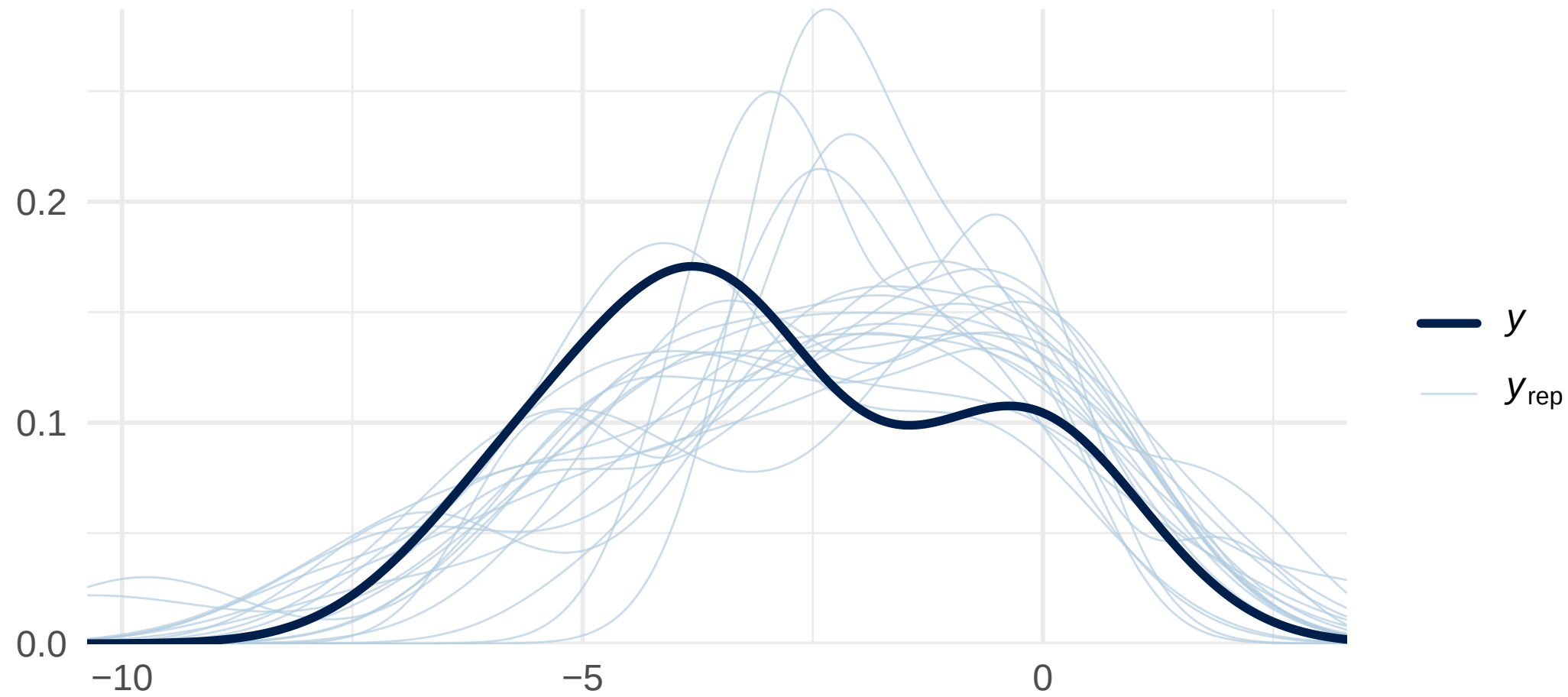

Posterior predictive check, using pp_check() from the bayesplot package. Density of the observed effect sizes (dark line) compared with 20 data sets simulated from the posterior predictive distribution of the fitted model (light lines). This checks how well the fitted model captures and reproduces the observed data.

5c) MCMC trace plots for mu and tau

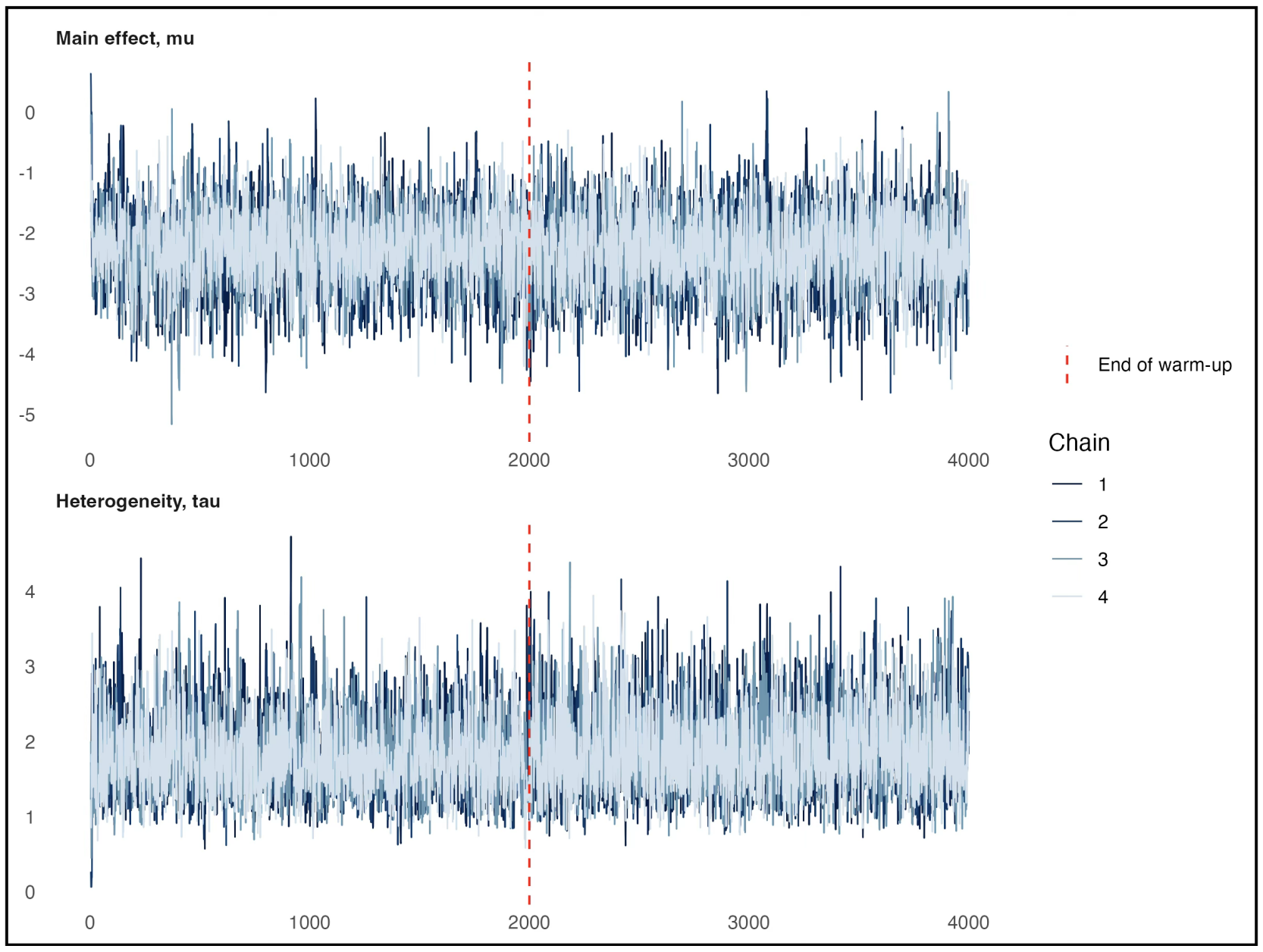

Traceplots for the pooled effect (mu) and between-study heterogeneity (tau), including the warm-up phase to the left of the dashed line. The four sampling chains are overlaid. Chains that overlap and show no trend indicate that the sampler settled on the same distribution from different starting points.

5d) Prior, observed effects and posterior when both priors change together

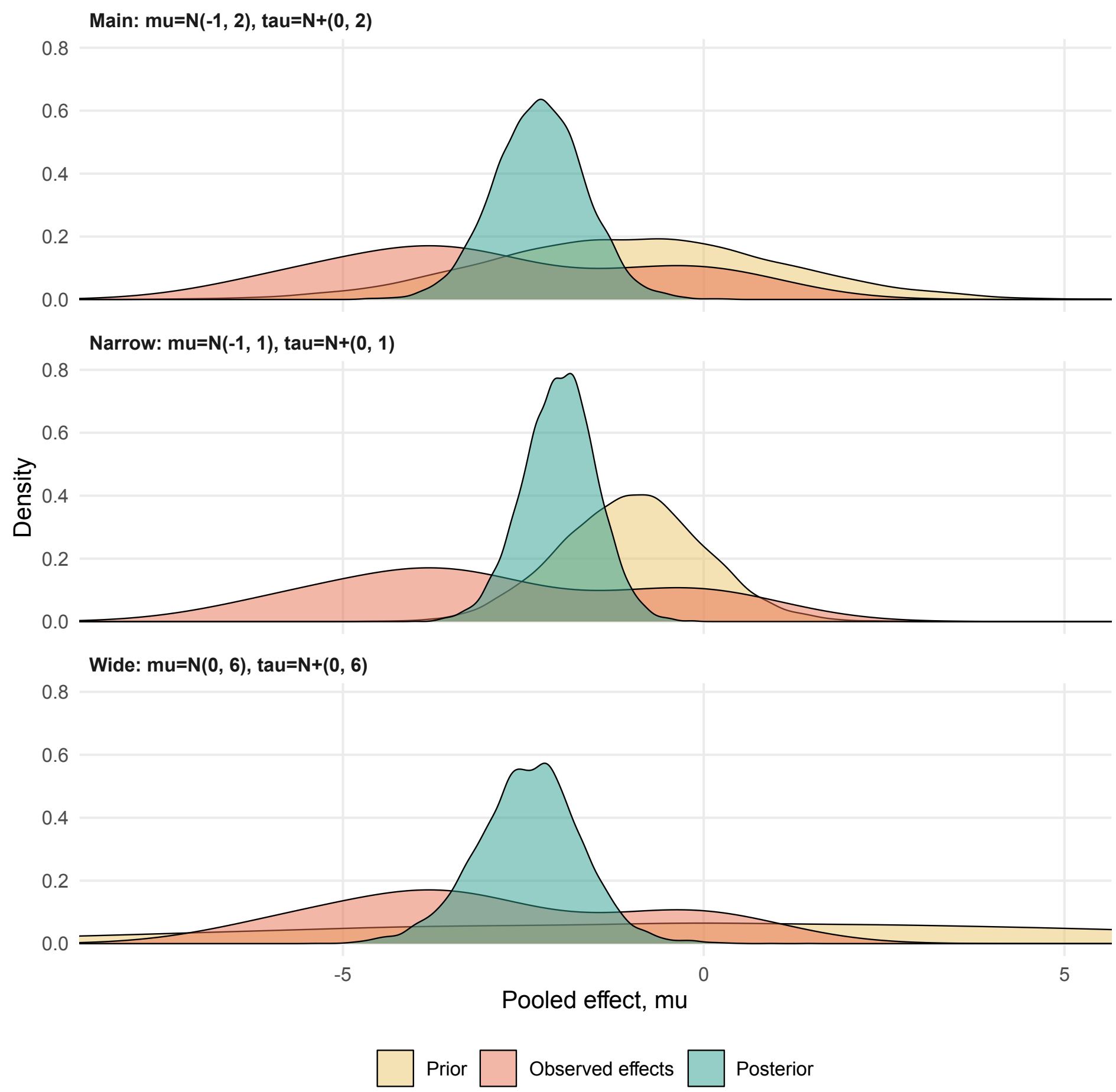

Prior, observed study effects and posterior distribution of the pooled effect (mu) under the three prior sets reported in Table 4, in which the effect prior and the heterogeneity prior change together. Each panel is scaled to unit area, so heights are comparable within a panel but not across panels. Because both priors move at once, a shift in the posterior cannot be attributed to either one.

5e) Varying only the effect prior

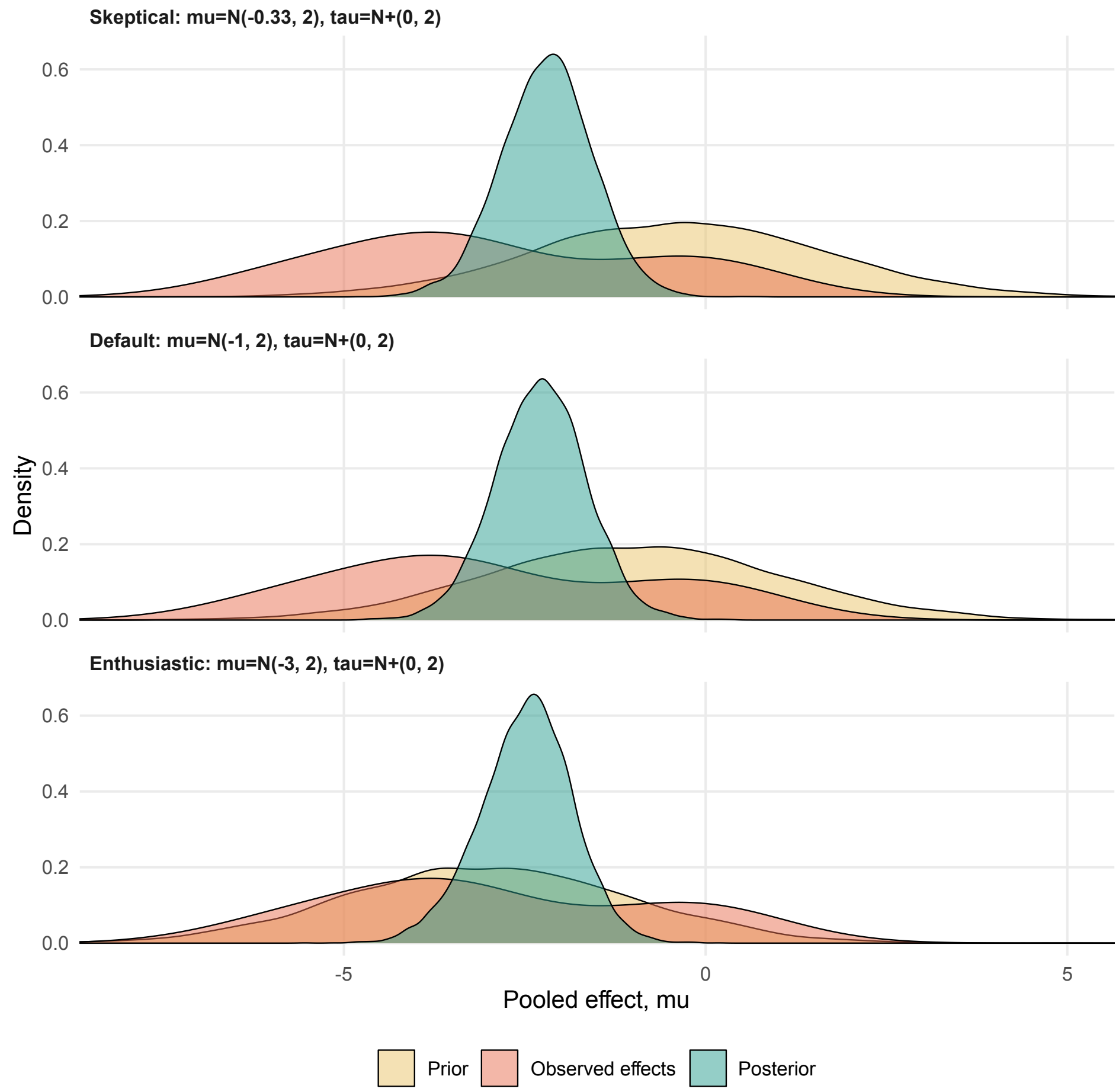

Prior, observed study effects and posterior distribution of the pooled effect (mu) when only the centre of the effect prior changes, from skeptical (closer to no effect) through the default to enthusiastic (a large effect). The heterogeneity prior is held at N+(2) throughout. The posterior moves very little, indicating that with twelve studies the observed data outweigh the effect prior.

5f) Varying only the heterogeneity prior

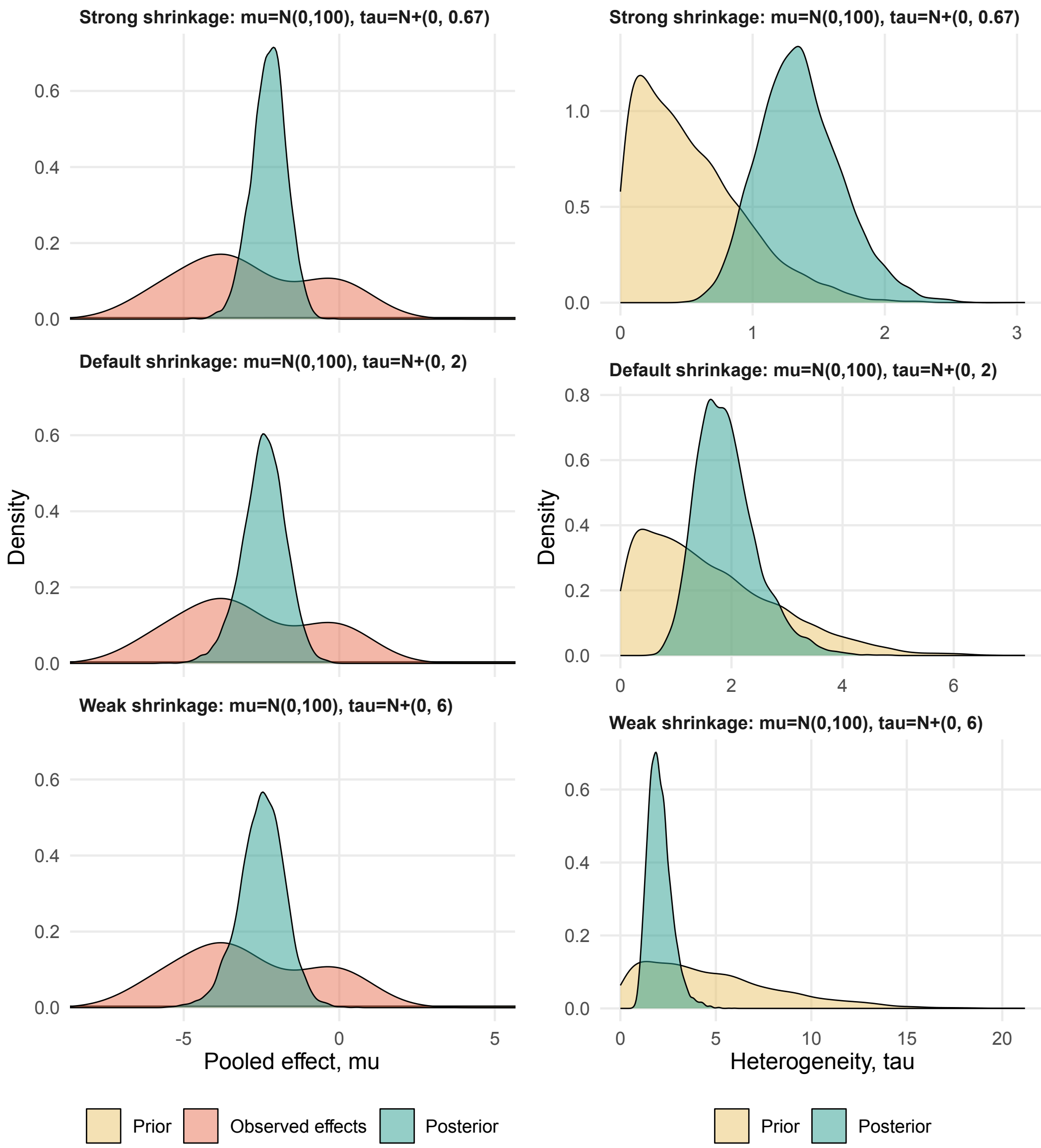

Prior and posterior distributions of the pooled effect (mu, left column) and of between-study heterogeneity (tau, right column) when only the width of the heterogeneity prior changes. The effect prior is held at Normal(0, 100), which is so wide that it appears as a flat line and contributes almost no information. A narrow heterogeneity prior limits how far individual study estimates may sit from the pooled mean, which pulls the pooled effect toward no effect; a wide one allows imprecise studies reporting large effects to pull it away.

5g) Funnel plot

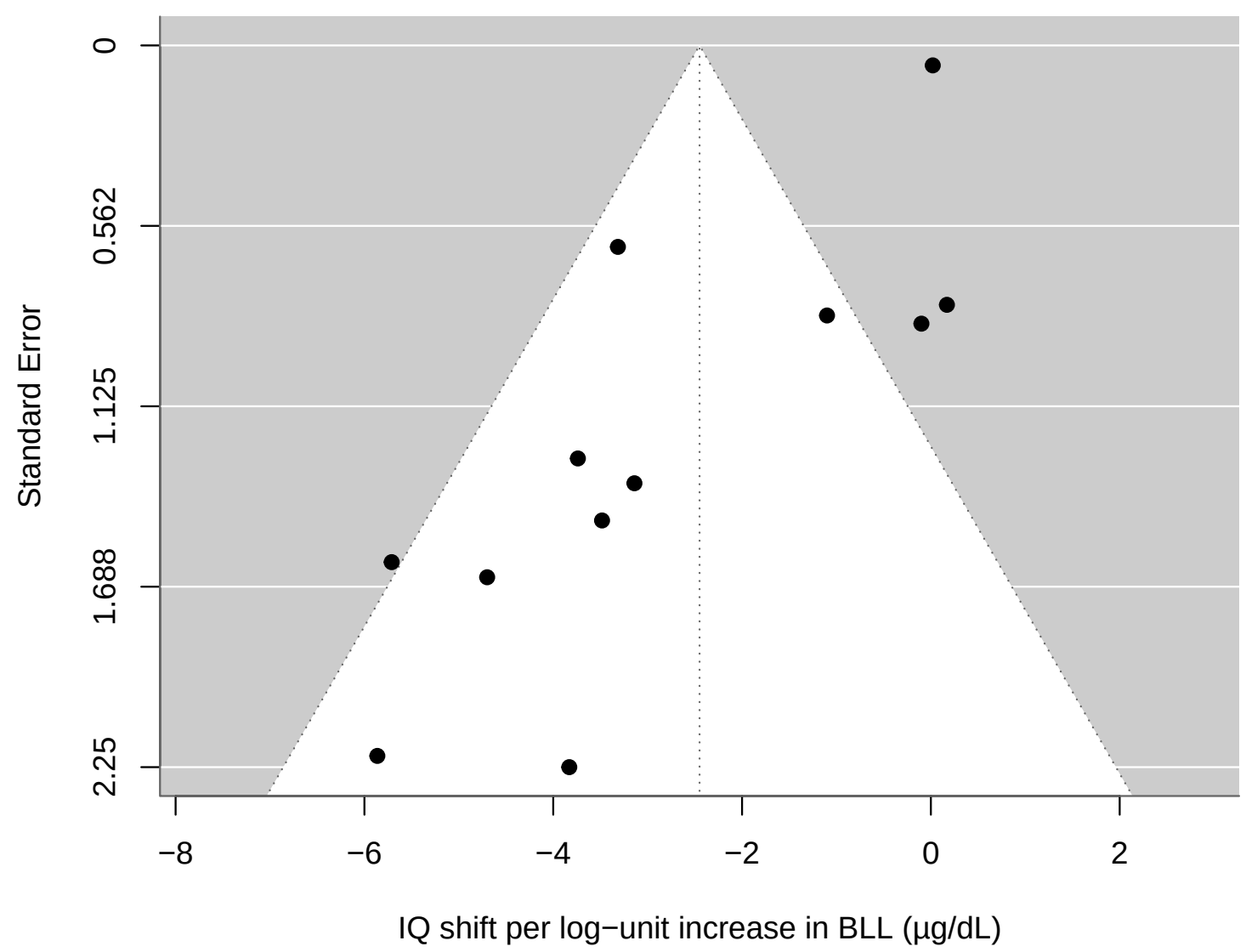

Funnel plot of the frequentist model. Each point is one study, plotted against its standard error, so imprecise studies sit lower. Asymmetry, meaning imprecise studies falling mostly on one side of the pooled estimate, is consistent with publication bias.
