# Supplementary material for: Quantifying uncertainty: a practical comparison of Bayesian and frequentist meta-analytic frameworks applied to lead exposure and children’s IQ
Source: BMC Med Res Methodol. 2026 Sep 24;26:209. doi: 10.1186/s12874-026-03010-z (PMC13602614; doi:10.1186/s12874-026-03010-z)
Supplement: Supplementary file 3 [file 12874_2026_3010_MOESM6_ESM.pdf]

Full model output for frequentist and Bayesian meta-analysis

This supplementary file contains the model code and output for both meta-analysis frameworks.

Frequentist meta-analysis, using {metafor}

Model code

```
rma_model <- rma(
  yi = beta_ln,
  sei = se_beta_ln,
  data = data_full,
  method = "REML"
)
```

Output

```
Random-Effects Model (k = 12; tau^2 estimator: REML)

  logLik  deviance       AIC       BIC      AICc
-24.2820   48.5640   52.5640   53.3598   54.0640

tau^2 (estimated amount of total heterogeneity): 3.3236 (SE = 2.0238)
tau (square root of estimated tau^2 value):      1.8231
I^2 (total heterogeneity / total variability):   84.97%
H^2 (total variability / sampling variability):  6.65

Test for Heterogeneity:
Q(df = 11) = 78.5314, p-val < .0001

Model Results:

estimate      se     zval    pval    ci.lb    ci.ub
 -2.4500  0.6371  -3.8453  0.0001  -3.6988  -1.2012  ***
```

```
---
Signif. codes:  0 '***' 0.001 '**' 0.01 '*' 0.05 '.' 0.1 ' ' 1
```

Bayesian meta-analysis, using {brms}

Setting priors

```
priors <- c(
  prior(normal(-1, 2), class = Intercept),
  prior(normal(0, 2), class = sd, lb = 0)
)
```

Model code

```
m.brm <- brm(
  beta_ln | se(se_beta_ln) ~ 1 + (1 | author_year),
  data = data,
  prior = priors,
  save_pars = save_pars(all = TRUE),
  chains = 4,
  iter = 4000
)
```

Model code: prior only (for prior predictive checks)

```
fitPrior <- brm(
  beta_ln | se(se_beta_ln) ~ 1 + (1 | author_year),
  data = data,
  prior = priors,
  sample_prior = "only",
  chains = 4,
  iter = 4000
)
```

Output main model

```
 Family: gaussian
  Links: mu = identity; sigma = identity
Formula: beta_ln | se(se_beta_ln) ~ 1 + (1 | author_year)
   Data: data (Number of observations: 12)
  Draws: 4 chains, each with iter = 4000; warmup = 2000; thin = 1;
         total post-warmup draws = 8000
```

```
Multilevel Hyperparameters:
~author_year (Number of levels: 12)
              Estimate Est.Error l-95% CI u-95% CI Rhat Bulk_ESS Tail_ESS
sd(Intercept)     1.88      0.53     1.02     3.08 1.00     2292     3519

Regression Coefficients:
          Estimate Est.Error l-95% CI u-95% CI Rhat Bulk_ESS Tail_ESS
Intercept    -2.28      0.64    -3.54    -1.04 1.00     1796     2504

Further Distributional Parameters:
      Estimate Est.Error l-95% CI u-95% CI Rhat Bulk_ESS Tail_ESS
sigma     0.00      0.00     0.00     0.00   NA       NA       NA

Draws were sampled using sampling(NUTS). For each parameter, Bulk_ESS
and Tail_ESS are effective sample size measures, and Rhat is the potential
scale reduction factor on split chains (at convergence, Rhat = 1).
```

Output prior only sampling

```
 Family: gaussian
  Links: mu = identity; sigma = identity
Formula: beta_ln | se(se_beta_ln) ~ 1 + (1 | author_year)
   Data: data (Number of observations: 12)
  Draws: 4 chains, each with iter = 4000; warmup = 2000; thin = 1;
         total post-warmup draws = 8000

Multilevel Hyperparameters:
~author_year (Number of levels: 12)
              Estimate Est.Error l-95% CI u-95% CI Rhat Bulk_ESS Tail_ESS
sd(Intercept)     1.59      1.19     0.06     4.43 1.00     5959     3547

Regression Coefficients:
          Estimate Est.Error l-95% CI u-95% CI Rhat Bulk_ESS Tail_ESS
Intercept    -1.00      2.01    -4.90     2.99 1.00    10977     5801

Further Distributional Parameters:
      Estimate Est.Error l-95% CI u-95% CI Rhat Bulk_ESS Tail_ESS
sigma     0.00      0.00     0.00     0.00   NA       NA       NA

Draws were sampled using sampling(NUTS). For each parameter, Bulk_ESS
and Tail_ESS are effective sample size measures, and Rhat is the potential
scale reduction factor on split chains (at convergence, Rhat = 1).
```
